# Supplementary material for: Limited Knowledge of Toxoplasmosis-Specific Preventive Behaviors in Pregnant Women: A Survey Study in Northern Italy
Source: Int J Environ Res Public Health. 2025 Mar 28;22(4):517. doi: 10.3390/ijerph22040517 (PMC12027400; doi:10.3390/ijerph22040517)
Supplement: Supplementary file 1 [file ijerph-22-00517-s001.zip › ijerph-3409557-supplementary.pdf]

Survey

Sociodemographic data

- How old are you?
- Nationality ? □ European □ non-European
- Schooling: □ Primary school □Secondary school □ University
- How many children do you have? How old are they? □ 1 □ 2 □ 3 □ 4 □ 5 □ 6 Age ___

Information related to your current pregnancy

- Date of last period:________
- Expected date of birth:_______
- Where is your pregnancy being managed?
  □ Academic maternity center
  □ Community
  □ First-level hospital
  □ Private healthcare professional

Questionnaire

- Have you received information on preventive measures during pregnancy?
  □ yes □ no
- When have you received this information?
  □ 1st antenatal appointment
  □ 1st scan
  □ at one antenatal appointment during pregnancy

- Who provided you such information?
  □ Healthcare professionals (gynecologist/midwife)
  □ Family members
  □ Friends
  □ Mass-media

- Were you already aware of such information before the pregnancy?
  □yes □no

- If the answer to the previous question is "YES", who provided you such information?
  □ Healthcare professionals (gynecologist/midwife)
  □ Family members
  □ Friends
  □ Mass-media

- Are you takingfolic acid?
  □ yes □ no

- If the answer to the previous question is 'YES,' when did you start takingfolic acid?
  □ before pregnancy
  □ following positive pregnancy test
  □ following 1st antenatal appointment

- Are you smoking during your pregnancy?
  □ no
  □ yes

- In your opinion, what is the recommended alcohol intake during pregnancy?
  □ It is forbidden to drink alcohol during pregnancy
  □ 1-2 glasses of wine/beer per week
  □ 1-2 glasses of wine/beer per month

- Have you ever heard of the following conditions? If yes, which one?

  ☐ Toxoplasmosis ☐Listeria ☐Parvovirus B19 ☐Down's Syndrome
  ☐Cytomegalovirus ☐Chickenpox ☐Rubella ☐HIV
  ☐Hepatitis C and B ☐Spina bifida ☐Streptococcus beta-Agalactiae
  ☐Syphilis ☐Measles ☐Fetal Alcohol Syndrome

- Which one of the following infections could be a problem if acquired in pregnancy?

  ☐ Toxoplasmosis ☐Listeria ☐Parvovirus B19 ☐Cytomegalovirus
  ☐Chickenpox ☐Rubella ☐HIV ☐Hepatitis C and B ☐Syphilis
  ☐Measles ☐Streptococcus beta-Agalactiae

- Which one of the following behaviours may help avoiding acquisition ofToxoplasmosis gondii infection?
  □ Avoiding kissing babies on the mouth
  □ Avoiding sharing glasses or cutlery with the child
  □ Washing your hand after touching the mouth or the nose of the child
  □ Takingfolic acid
  □ Washing raw vegetables and fruits before eating them
  □ Washing hands after changing diapers
  □ Avoid eating raw meat
  □ Washing hands after garden work
  □ Avoid contact with dogs
  □ Avoid contact with cats
